# Ten-Year Differences in Nutritional Status and Obesity-Related Risk Factors in Polish Preschool Children

**DOI:** 10.3390/children10040636

**Published:** 2023-03-28

**Authors:** Marcela Zembura, Paweł Lula, Paweł Matusik

**Affiliations:** 1 Department of Pediatrics, Pediatric Obesity and Metabolic Bone Diseases, Faculty of Medical Sciences in Katowice, Medical University of Silesia, 40-055 Katowice, Poland; 2 Department of Computational Systems, Cracow University of Economics, 31-510 Kraków, Poland

**Keywords:** preschool, children, obesity

## Abstract

The aim of this study was to assess ten-year differences in nutritional statuses and obesity prevalences between populations of preschool children from Katowice, Poland, examined in 2007 and 2017, and to determine factors associated with overweight and obesity in preschool children. A cross-sectional questionnaire was conducted among parents and legal guardians of 276 preschool children in 2007 and 259 preschool children in 2017. Basic anthropometric measurements were performed. Overall, the prevalence of overweight and obesity among our sample of Polish preschool children (median age 5.25 year) was 16.82%, whereas 4.49% of children were obese. No significant differences in the number of overweight and obese children were observed when comparing the years 2017 to 2007. Overall body mass index (BMI) z-score was significantly lower in this group of children from 2017. However, median values of the BMI z-score were higher in two of the weight categories (overweight and obesity) in 2017. The child’s BMI z-score was positively correlated with birth weight (r = 0.1, *p* < 0.05). The BMI z-score was positively correlated with maternal BMI, paternal BMI, and maternal pregnancy weight gain, r = 0.24 *p* < 0.01; r = 0.16 *p* < 0.01; r = 0.12 *p* < 0.05, respectively. A decrease in overweight and obesity prevalence over the past decade and higher median values of BMI z-scores in the group of children with excessive weight in 2017 were observed. Birth weight, maternal BMI, paternal BMI, and maternal pregnancy weight gain all correlate positively with a child’s BMI z-score.

## 1. Introduction

According to World Health Organization (WHO), in the year 2019, approximately 38.2 million children below the age of five were overweight or obese [1]. Obesity affects not only children from high-income countries but is also rising in low- and middle-income countries [1]. Despite the implementation of numerous obesity prevention programs, the prevalence of overweight and obesity among preschool children is rising on each of the continents and poses a serious public health challenge [2].

Obesity in children leads to a wide variety of comorbid conditions and complications. Childhood obesity is linked to various metabolic complications, including dyslipidemia, decreased insulin sensitivity, increased circulating insulin levels, glucose intolerance, type 2 diabetes mellitus, and metabolic syndrome [3]. Several studies have shown that the metabolic syndrome may be present in 30% of obese children and adolescents [4]. Childhood obesity is linked to the occurrence of nonalcoholic fatty liver disease (NAFLD), obstructive sleep apnea, asthma, tibia vera, and psychosocial problems [5]. Childhood obesity is also linked to higher prevalence of disability and obesity in adulthood and premature death [1]. According to the Muscatine Study, being overweight in childhood might predict the occurrence of coronary artery calcification in young adulthood [6].

There are various factors linked to the occurrence of childhood obesity; however, the role of some of them is still unclear. Although the vast majority of childhood obesity cases are exogenous, a small proportion may have endogenous causes (genetic syndromes, hormonal disorders, and monogenetic disorders) [7]. Parental body mass index (BMI) might be a predictor of obesity during childhood, which indicates a genetic effect [8]. Nevertheless, genetic factors may not be the only contribution since children’s food choices and eating behaviors are learned from parents at very young ages [9].

A wide range of factors associated with childhood obesity were identified, including maternal, paternal, gestational, and postnatal factors (such as birth weight, type of feeding, rapid infant growth, sleep duration, and screen time) [10]. Both maternal pre-pregnancy BMI and paternal BMI, as well as gestational weight gain, gestational diabetes, smoking during pregnancy, and type of birth were found to be associated with childhood obesity [11,12,13,14,15]. A study performed in a population-based sample of 51,505 children reported that 43.7% of children large for gestational age at birth were overweight/obese in adolescence [16]. Various studies indicated that breastfeeding has a protective effect on obesity risk as breast-fed infants gain body mass slower than formula-fed infants [17,18,19]. According to the study by Iguacel et al., rapid infant weight gain during the first 6 months of life was found to be an independent predictor of excessive weight in children aged 6 [20]. Several studies reported an association between short sleep duration and increased risk of overweight and obesity in childhood [21,22]. A systematic review regarding screen time in children below 3 years of age indicated that screen time correlated with the BMI of the child [23].

Recent studies suggest that in children with obesity, most of their excess weight is gained in their first 5 years of age [24]. Therefore, the early years of childhood are important in terms of the development of obesity and the implementation of prevention and intervention programs. However, despite the implementation of numerous intervention programs regarding the pediatric population, according to the review by Flynn et al., only 6% concerned the 0–5-year age range [2].

Regarding the rapid rise in prevalence of obesity in preschool children, its association with severe comorbidities, obesity in adulthood, and the lack of consistent data regarding factors leading to childhood obesity, further research on this topic is required. Regarding the lack of studies assessing 10-year differences in preschool children’s nutritional status, we have identified a research gap. The aim of this study was to assess the differences in nutritional status and obesity prevalence that occurred between populations of preschool children from Katowice, Poland, examined in 2007 and 2017, and to determine maternal, paternal, gestational, and infantile factors associated with overweight and obesity in preschool children.

## 2. Materials and Methods

The study was conducted using data from a cross-sectional questionnaire conducted among parents and legal guardians of children attending preschool in Katowice, Poland, in the years 2007 (g-2007) and 2017 (g-2017). Basic anthropometric measurements were performed in both groups (g-2007 and g-2017).

All of the preschools located in Katowice, Poland, in 2007 were invited to participate in our study. The management of 11 preschools in Katowice, Poland, agreed to take part in the study. Invitations regarding participation in the study were sent to all of the parents/legal guardians of children from 11 preschools. The questionnaire was handed over to the parents/legal guardians of the children who agreed to be involved in the study. Afterwards, written consent concerning anthropometric measurements was sent to all of the parents/legal guardians. Anthropometric measurements were performed after receiving informed consent from the parents/legal guardians. The same 11 preschools from Katowice, Poland, were included in our study in 2017. Therefore, the children who were recruited were from the same neighborhoods and had similar socioeconomic backgrounds.

The questionnaire was divided into 10 parts concerning: general questions regarding children and parents; pregnancy and infancy; kindergarten; eating habits; physical activity and play; screen time; family habits of buying food; eating habits of the family; social life; and health. However, the aim of this study was to evaluate parts of the questionnaire regarding general questions, such as those about pregnancy, infancy, and kindergarten. The questionnaire was performed twice: in 2007 (g-2007) and in 2017 (g-2017). The study group consisted of 535 children: 276 (g-2007) and 259 (g-2017). The median age in the g-2007 was 5.25 (4.17; 6.08) years, and in the g-2017, it was 5.5 (4.5; 6.29) years.

### 2.1. Anthropometric Measurements

Weight was measured in light clothes and without shoes to the nearest 0.1 kg with an electronic scale, and height was measured barefoot to the nearest 0.1 cm using a wall-mounted stadiometer. BMI was defined as weight divided by height squared (kg/m^2^). BMI was converted to age- and sex-specific z-scores using WHO AnthroPlus, which is the global application of the WHO Reference [25]. Children were divided into 4 weight categories (underweight, normal, overweight, and obese) according to WHO references [1,26].

### 2.2. Ethical Considerations

Ethical review and approval were waived for this study due to the fact that it was conducted using a questionnaire. Only basic anthropometric measurements, which are routinely assessed during examination, were measured.

Written informed consent was obtained from the parents or legal guardians of all children prior to participation in the study.

### 2.3. Statistical Analysis

The preliminary analysis of variables was performed by calculating descriptive statistics (mean, standard deviation, median, quartiles, minimum, maximum, range, skewness, and kurtosis). Furthermore, for every variable, the probability density function was estimated. It was carried out separately for the years 2007 and 2017.

The analysis of relationships between qualitative variables was performed using the chi-square test. For verification of relationships between qualitative and quantitative variables, analysis of variance (ANOVA) was performed. Assumptions of ANOVA were verified with the use of Levene’s test (homogeneity of variances) and the Shapiro-Wilk test (normality of residuals). If assumptions were fulfilled, then classical ANOVA analysis was conducted, and the Tukey Honest Significant Differences test was used for testing the significance of differences between average values of a quantitative variable calculated for groups corresponding to particular values of a qualitative variable. If the assumptions of ANOVA were not fulfilled, then the Kruskal-Wallis rank sum test was used for verification of the significance of the relationship between variables and Dunn’s test—for testing the significance of differences between average values of a given quantitative variable in every pair of groups. 

To compare the distribution of a quantitative variable across two groups, the Kolmogorov-Smirnov test was performed. Since the majority of data were not normally distributed, quantitative data were described as medians and interquartile ranges (IQR). A *p* value of <0.05 was considered statistically significant. The analysis was conducted with the use of Microsoft Excel and the R computing system. 

## 3. Results

The study was conducted on a sample of 535 preschool children, of whom 49.72% were girls. The median age of the preschool child was 5.25 (4.25; 6.17) years. 

### 3.1. Nutritional Status

**Figure 1:**
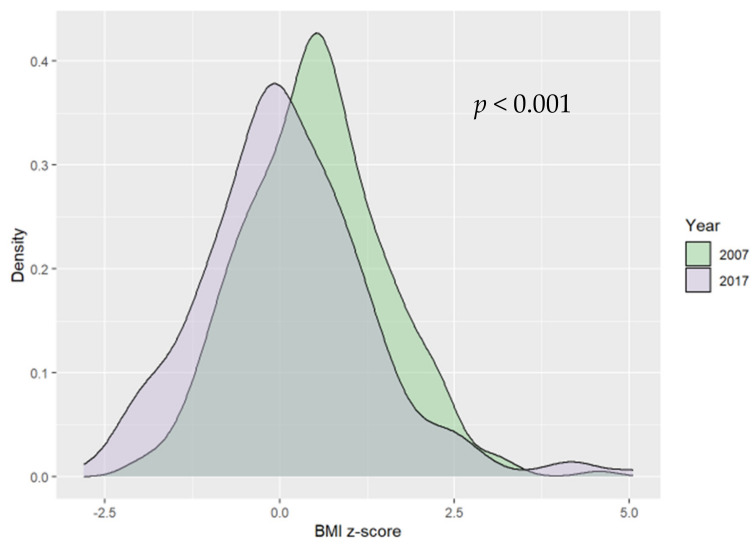
Ten−year differences in body mass index (BMI) z−scores.

The number of children in each weight category was presented in Table 1. Overall, 16.82% (*n* = 90) of the study group was overweight or obese, of which 44.44% (*n* = 40) were girls. A statistically significant difference between groups from 2007 and 2017 in terms of BMI z-score was observed; BMI z-score was significantly lower in 2017 than in 2007 (0.02 SD vs. 0.48 SD, respectively) (see Figure 1).

**Figure 2:**
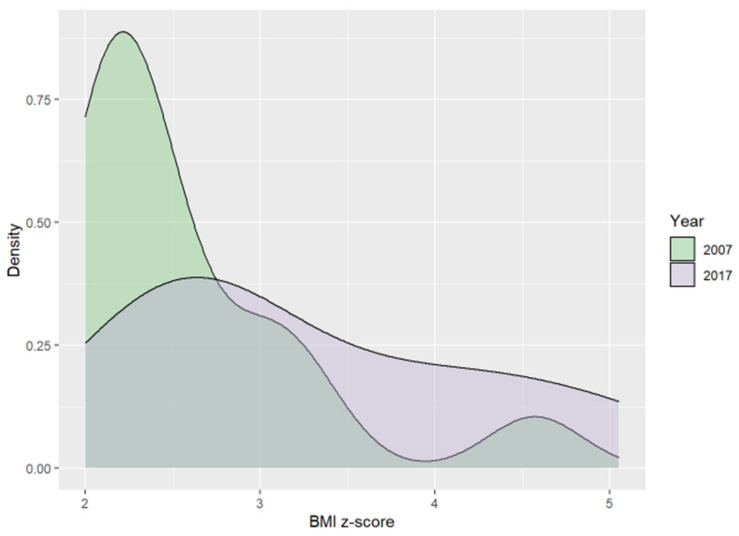
Ten-year differences in BMI z-scores in obese children.

There were no significant differences in the number of overweight and obese children between 2017 and 2007. However, visible trends included a decrease in overweight and obesity prevalence over the past decade (Table 1). The median values of the BMI z-score were higher in two of the weight categories (overweight and obesity) in 2017. However, the differences were not statistically significant. Figure 2 shows ten-year differences in BMI z-score in obese children; the median value of BMI z-score in this weight group in 2017 was 2.9 vs. 2.33 in 2007.

In the other two weight categories (underweight and normal), median BMI z-score values were lower in 2017, but the difference was statistically significant only in the normal weight group (*p* < 0.001). With regard to the normal weight group, values of the BMI z-score were significantly lower in 2017, indicating improvement in nutritional status in this weight category (see Table 2).

### 3.2. Factors Associated with Excessive Weight

Analysis of the data from questionnaires indicated statistically significant differences between groups in terms of maternal and paternal ages. The obtained results show that the age of parents of children in g-2017 was significantly higher compared to g-2007. 

Children’s age, maternal BMI, and paternal BMI were higher in children from g-2017, whereas birth weight and maternal pregnancy weight gain were lower in g-2017. However, the differences in terms of age, childbirth weight, paternal and maternal BMIs, and the mother’s pregnancy weight gain between the children from g-2007 and g-2017 were not statistically significant. 

Table 3 shows factors associated with excessive weight in children overall and by the years in which the study was conducted. 

The child’s BMI z-score was positively correlated with maternal BMI, maternal pregnancy weight gain, paternal BMI, and the child’s birth weight. Spearman’s correlation coefficients between BMI z-score and maternal weight, maternal pregnancy weight gain, paternal weight, and the child’s birth weight are shown in Table 4.

The percent of fathers with vocational and college education was higher in the group of children with excessive weight, whereas the percent of fathers with primary and university education was lower in the group of children with overweight/obesity. The percent of mothers with primary, vocational, and college education was higher in the group of children with excessive weight, with a higher percent of mothers with university education in the normal weight group (Table 5).

However, differences in parental education between children with normal weight and overweight/obese children were not statistically significant (*p* > 0.05), probably due to the low number of children in the excessive weight group. 

## 4. Discussion

This study investigated ten-year differences in nutritional statuses and obesity prevalences that occurred between populations of preschool children from Katowice, Poland, examined in 2007 and 2017, and determined maternal, paternal, gestational, and infantile factors associated with excess weight in preschool children.

Among our sample of Polish preschool children (median age 5.25 year), 16.82% were overweight/obese, and 4.49% were obese. A significant difference in the median BMI z-score from 2007 to 2017 was observed. The median BMI z-score was significantly lower in 2017. The median values of the BMI z-score were higher in two of the weight categories (overweight and obesity) in 2017. The occurrence of extreme values of the BMI z-score in a group of children with obesity in 2017 was observed. In the normal weight category, median BMI z-score values were significantly lower in 2017. There were no significant differences in the number of overweight and obese children between 2017 and 2007. However, visible trends included a decrease in overweight and obesity prevalence. Differences in terms of parental age between populations examined in 2007 and 2017 were observed. The BMI z-score was positively correlated with maternal BMI, maternal pregnancy weight gain, paternal BMI, and birth weight. However, it must be noted that the correlations were weak.

The prevalence of overweight and obesity in our study is consistent with previous studies regarding preschool children. In one study estimating the global prevalence of overweight and obesity among preschool children from 1990–2010 and projecting its prevalence in 2015 and 2020, in 2010, 11.7% of children from developed countries were overweight/obese, and the projected number of children with excessive weight in developed countries in 2015 and 2020 was 12.9% and 14.1%, respectively [27]. In a cross-sectional Polish study involving preschool children, the prevalence of obesity differed depending on the definition and ranged from 19.9% (based on body fat percentage) to 4.4% (based on BMI) [28]. According to the study by Baran et al. regarding preschool children from south-eastern Poland, the prevalence of excessive body mass (overweight and obesity) was slightly lower than in our study (12%), but the number of obese children was more similar to the results of our study (3.3%) [29]. In the systematic review and meta-regression, which aimed to analyze the prevalence of overweight and obesity among European children aged 2–7 years from 2006 to 2016 using International Obesity Task Force (IOTF) definition criteria, the pooled prevalence of excessive weight in Europe was 17.9% (95% CI: 15.8–20.0) and the pooled prevalence of obesity was 5.3% (95% CI: 4.5–6.1) [30].

Overall, the BMI z-score was significantly lower in a group of children in 2017; however, among the children with overweight and obesity, the BMI z-scores were higher in 2017 than in 2007. Moreover, from the overview of Table 2, it is observable that in 2017, the values of the upper quartile of the BMI z-score in the group of obese children were higher, indicating the occurrence of a tendency towards higher values of the BMI z-score in obese children. Moreover, although no statistically significant differences in terms of prevalence of normal weight as well as excessive weight were observed, a tendency regarding a decrease in overweight and obesity rates and an increase in the prevalence of normal weight from 2007 to 2017 was visible. Results regarding differences in percentages of children in different weight categories (normal, overweight, and obesity) between 2007 and 2017 are similar to previous studies. In the study by Żegleń et al., which assessed differences in excessive weight prevalence in Polish preschool children between the years 2008 and 2018, the number of children with normal BMI was higher, whereas a decrease was noted in the number of children with overweight and obesity between 2008 and 2018 [31]. Furthermore, the study by Koebnick et al., which included a Southern California pediatric population, showed that the prevalence of excessive weight declined between 2008 and 2013 [32].

In our study, the medians of maternal and paternal age among preschool children were significantly higher in 2017 than in 2007. The differences can be justified by the fact that in the developed world, the average age at which people have children is increasing rapidly [33].

The results of our study regarding the association between child BMI z-score and birth weight match previous studies. In a study by Olson et al., infant birth weight was positively associated with a higher risk of obesity in children aged 4 [34]. According to the meta-analysis by Yu et al., birth weight above 4000 g was associated with an increased risk of later obesity, whereas no association was found in the group of children with normal and low birth weight [35]. Results of our study indicated that preschool-aged children’s BMI is correlated with parental weight status. These results are in line with the previous cross-sectional study regarding Polish preschool children; in this study, children who had obese mothers had a higher risk of excess weight (adj. OR = 2.66, 95% CI: 1.44–4.89); the risk of excessive weight in children when both parents were obese was even higher (adj. OR = 5.31, 95% CI: 2.48–11.4) [28]. In a study by Olson et al., there was no significant association between overall maternal change of weight between 1 year and 2 years postpartum and children’s weight; however, children of mothers who had lost weight during this period were less likely (*p* < 0.05) to have excessive weight than children whose mother’s weight was stable or had gained weight [34]. The results concerning the positive correlation between BMI z-score of a child and maternal pregnancy weight gain obtained from our study are consistent with the study by Rossem et al., which indicated that children whose mothers experienced excessive pregnancy weight gain had a higher prevalence of overweight (odds ratio [OR] 1.20; 95% confidence interval [CI], 0.99 to 1.46) and a higher BMI z-score throughout childhood [36]. This study did not identify the association between parental education level and excessive weight in preschool children. Some other studies, as well as our study, have not found statistically significant differences regarding the association between parental education level and the occurrence of excessive weight in preschool children [37,38].

Developmental factors predisposing to overweight and obesity at age 5 include the mother’s preconceptional BMI, mothers smoking 1–12 cigarettes/day during the 1st trimester, a smaller number of children at home, the child’s high birth weight, the female sex of the child, the pattern of early growth, and nutrition [39,40]. Obesity risk was found to be strongly related to rapid growth during infancy, an effect linked to nutrition [41]. Various studies indicated that breastfeeding has a protective effect on obesity risk [18,42]. In formula-fed infants, early protein intake was found to be 70% greater than in infants who were breast-fed this might be associated with an increased infant growth rate and therefore increase the risk of later obesity [40]. The role of diet in childhood obesity has been the subject of numerous studies. However, their results regarding associations between energy intake [43,44], fat and carbohydrate intake [43,45] and obesity prevalence are conflicting. Although various studies indicated that physical activity might reduce the risk of childhood obesity [46], intervention studies regarding physical activity in preschool children were unsuccessful [40].

This study has several limitations that warrant consideration. First, the sample does not represent the entire population of Poland since it only includes respondents from one city. Second, there is a lack of data regarding response rate.

Nevertheless, despite these limitations, this study provides data regarding ten-year differences in nutritional status and obesity prevalence between years 2007 and 2017 and results regarding risk factors for overweight/obesity in preschool-aged children. The results of our study might be useful for public health in terms of implementation of prevention and treatment programs among preschool children. Moreover, results could be used to identify vulnerable subgroups of children and implement prevention programs in order to avoid the occurrence of obesity and its comorbid conditions in children and therefore prevent adverse health outcomes associated with obesity in adulthood.

Further studies regarding nutritional status among preschool children should be conducted in order to assess the COVID-19 pandemic’s impact on weight status.

## 5. Conclusions

Among our samples of Polish preschool children, 16.82% were overweight/obese and 4.49% were obese. Overall, the BMI z-score was significantly lower in the group of children from 2017; however, among the children with overweight and obesity, the BMI z-scores were higher in 2017 than in 2007. The BMI z-score was positively correlated with maternal BMI, maternal pregnancy weight gain, paternal BMI, and birth weight.

## Figures and Tables

**Table 1:** Number of children in four weight categories (underweight, normal, overweight, and obesity).

	Underweight	Normal	Overweight	Obesity
2007	0.72%	78.99%	15.22%	5.07%
(*n* = 276)	(*n* = 2)	(*n* = 218)	(*n* = 42)	(*n* = 14)
2017	3.09%	83.78%	9.27%	3.86%
(*n* = 259)	(*n* = 8)	(*n* = 217)	(*n* = 24)	(*n* = 10)

**Table 2:** BMI z-score in 4 weight categories (underweight, normal, overweight, and obesity). Values are medians (lower quartile and upper quartile).

	Underweight	Normal	Overweight	Obesity
2007	−1.63	0.32	1.53	2.33
	(−1.85; −1.4)	(−0.33; 0.7)	(1.29; 1.87)	(2.14; 2.83)
2017	−2.2	−0.11	1.57	2.9
	(−2.89; −2.14)	(−0.67; 0.43)	(1.22; 1.76)	(2.47; 3.97)
*p* value	NS	*p* < 0.001	NS	NS

Abbreviations: NS—not significant.

**Table 3:** Factors associated with excessive weight, overall and by year.

	Overall	2007	2017	*p* Value
N	535	276	259	NA
Age (years)	5.25 (4.25; 6.17)	5.25 (4.17; 6.08)	5.5 (4.5; 6.29)	NS
Sex (M/F)	246/289	126/150	120/139	NS
Birth weight (g)	3350 (3000; 3650)	3400 (3053; 3650)	3315 (3010; 3620)	NS
Maternal age (years)	33 (30; 37)	33 (30; 35.25)	34 (30; 38)	*p* < 0.05
Paternal age (years)	35 (32; 39)	34 (31; 38)	36 (33; 39)	*p* < 0.05
Maternal BMI (kg/m^2^)	22.31 (20.3; 24.91)	22.23 (20.2; 24.96)	22.38 (20.45; 24.77)	NS
Paternal BMI (kg/m^2^)	26.12 (24.22; 28.37)	25.92 (24.22; 28.4)	26.47 (24.22; 28.36)	NS
Maternal pregnancy weight gain (kg)	14 (10; 18)	15 (10.75; 18)	14 (10; 20)	NS

Abbreviations: NA—not applicable; NS—not significant. Values are described as medians and interquartile ranges.

**Table 4:** Correlation between the BMI z-score, maternal BMI, maternal pregnancy weight gain, paternal BMI, and the child’s birth weight.

	Maternal BMI	Maternal Pregnancy Weight Gain (kg)	Paternal BMI	Birth Weight (kg)
BMI z-score	r = 0.24	r = 0.12	r = 0.16	r = 0.1
	*p* < 0.01	*p* < 0.05	*p* < 0.01	*p* < 0.05

**Table 5:** Parental education in two weight categories of children (normal and excessive weight).

		**Normal**	**Overweight/Obesity**
Paternal education level	primary	2.95% (*n* = 12)	2.67% (*n* = 2)
	vocational	21.13% (*n* = 86)	26.67% (*n* = 20)
	college	34.15% (*n* = 139)	42.67% (*n* = 32)
	university	41.77% (*n* = 170)	28% (*n* = 2)
Maternal education level	primary	2.31% (*n* = 10)	4.55% (*n* = 4)
	vocational	10.62% (*n* = 46)	13.64% (*n* = 12)
	college	35.57% (*n* = 154)	42.05% (*n* = 37)
	university	51.5% (*n* = 223)	39.77% (*n* = 35)

## Data Availability

The data presented in this study are available upon request from the corresponding author.

## References

[1] World Health Organization: Obesity and Overweight. http://www.who.int/.

[2] Flynn M.A.T., McNeil D.A., Maloff B., Mutasingwa D., Wu M., Ford C., Tough S.C. (2016). Reducing obesity and related chronic disease risk in children and youth: A synthesis of evidence with ‘best practice’ recommendations. Obes. Rev..

[3] Steinberger J., Moran A., Hong C.-P., Jacobs D.R., Sinaiko A.R. (2001). Adiposity in childhood predicts obesity and insulin resistance in young adulthood. J. Pediatr..

[4] Cook S., Weitzman M., Auinger P., Nguyen M., Dietz W.H. (2003). Prevalence of a metabolic syndrome phenotype in adolescents: Findings from the third National Health and Nutrition Examination Survey, 1988–1994. Arch Pediatr. Adolesc. Med..

[5] Daniels S.R. (2009). Complications of obesity in children and adolescents. Int. J. Obes..

[6] Mahoney L.T., Burns T.L., Stanford W., Thompson B.H., Witt J.D., Rost C.A., Lauer R.M. (1996). Coronary risk factors measured in childhood and young adult life are associated with coronary artery calcification in young adults: The muscatine study. J. Am. Coll. Cardiol..

[7] Brown C.L., Halvorson E.E., Cohen G.M., Lazorick S., Skelton J.A. (2015). Addressing Childhood Obesity: Opportunities for Prevention. Pediatr. Clin. N. Am..

[8] Pérusse L., Bouchard C. (1999). Role of genetic factors in childhood obesity and in susceptibility to dietary variations. Ann. Med..

[9] Birch L.L., Fisher J.O. (1998). Development of eating behaviors among children and adolescents. Pediatrics.

[10] Larqué E., Labayen I., Flodmark C.-E., Lissau I., Czernin S., Moreno L.A., Pietrobelli A., Widhalm K. (2019). From conception to infancy—Early risk factors for childhood obesity. Nat. Rev. Endocrinol..

[11] Leonard S.A., Petito L.C., Rehkopf D.H., Ritchie L.D., Abrams B. (2016). Weight gain in pregnancy and child weight status from birth to adulthood in the United States. Pediatr. Obes..

[12] Gaillard R., Steegers E.A., Duijts L., Felix J.F., Hofman A., Franco O.H., Jaddoe V.W. (2014). Childhood cardiometabolic outcomes of maternal obesity during pregnancy: The Generation R Study. Hypertension.

[13] Lawlor D.A., Lichtenstein P., Långström N. (2011). Association of maternal diabetes mellitus in pregnancy with offspring adiposity into early adulthood: Sibling study in a prospective cohort of 280 866 men from 248 293 families. Circulation.

[14] Oken E., Levitan E.B., Gillman M.W. (2008). Maternal smoking during pregnancy and child overweight: Systematic review and meta-analysis. Int. J. Obes..

[15] Mueller N.T., Whyatt R., Hoepner L., Oberfield S., Dominguez-Bello M.G., Widen E.M., Hassoun A., Perera F., Rundle A. (2015). Prenatal exposure to antibiotics, cesarean section and risk of childhood obesity. Int. J. Obes..

[16] Geserick M., Vogel M., Gausche R., Lipek T., Spielau U., Keller E., Pfäffle R., Kiess W., Körner A. (2018). Acceleration of BMI in Early Childhood and Risk of Sustained Obesity. N. Engl. J. Med..

[17] Weng S., Redsell S., Swift J.A., Yang M., Glazebrook C.P. (2012). Systematic review and meta-analyses of risk factors for childhood overweight identifiable during infancy. Arch. Dis. Child..

[18] Arenz S., Rückerl R., Koletzko B., Von Kries R. (2004). Breast-feeding and childhood obesity—A systematic review. Int. J. Obes. Relat. Metab. Disord..

[19] Rogers S.L., Blissett J. (2017). Breastfeeding duration and its relation to weight gain, eating behaviours and positive maternal feeding practices in infancy. Appetite.

[20] Iguacel I., Escartín L., Fernández-Alvira J.M., Iglesia I., Labayen I., Moreno L.A., Samper M.P., Rodríguez G., On behalf of the CALINA study group (2018). Early life risk factors and their cumulative effects as predictors of overweight in Spanish children. Int. J. Public Health.

[21] Baird J., Hill C.M., Harvey N.C., Crozier S., Robinson S.M., Godfrey K.M., Cooper C., Inskip H. (2016). The SWS Study Group Duration of sleep at 3 years of age is associated with fat and fat-free mass at 4 years of age: The Southampton Women’s Survey. J. Sleep Res..

[22] Cespedes E.M., Hu F.B., Redline S., Rosner B., Gillman M.W., Rifas-Shiman S.L., Taveras E.M. (2016). Chronic insufficient sleep and diet quality: Contributors to childhood obesity. Obesity.

[23] Duch H., Fisher E.M., Ensari I., Harrington A. (2013). Screen time use in children under 3 years old: A systematic review of correlates. Int. J. Behav. Nutr. Phys. Act..

[24] Gardner D.S.L., Hosking J., Metcalf B.S., Jeffery A.N., Voss L.D., Wilkin T.J. (2009). Contribution of Early Weight Gain to Childhood Overweight and Metabolic Health: A Longitudinal Study (EarlyBird 36). Pediatrics.

[25] Growth Reference Data for 5–19 Years. https://www.who.int/tools/growth-reference-data-for-5to19-years/application-tools.

[26] World Health Organization Reference Curves. http://ebook.ecog-obesity.eu/chapter-growth-charts-body-composition/world-health-organization-reference-curves/.

[27] de Onis M., Blössner M., Borghi E. (2010). Global prevalence and trends of overweight and obesity among preschool children. Am. J. Clin. Nutr..

[28] Matłosz P., Wyszyńska J., Asif M., Szybisty A., Aslam M., Mazur A., Herbert J. (2021). Prevalence of Overweight, Obesity, Abdominal Obesity, and Obesity-Related Risk Factors in Polish Preschool Children: A Cross-Sectional Study. J. Clin. Med..

[29] Baran J., Weres A., Czenczek-Lewandowska E., Łuszczki E., Sobek G., Pitucha G., Leszczak J., Mazur A. (2019). Early Eating Patterns and Overweight and Obesity in a Sample of Preschool Children in South-East Poland. Int. J. Environ. Res. Public Health.

[30] Garrido-Miguel M., Oliveira A., Cavero-Redondo I., Álvarez-Bueno C., Pozuelo-Carrascosa D.P., Soriano-Cano A., Martínez-Vizcaíno V. (2019). Prevalence of Overweight and Obesity among European Preschool Children: A Systematic Review and Meta-Regression by Food Group Consumption. Nutrients.

[31] Żegleń M., Kryst Ł., Kowal M., Woronkowicz A., Sobiecki J. (2020). Changes in the prevalence of overweight/obesity and adiposity among pre-school children in Kraków, Poland, from 2008 to 2018. J. Biosoc. Sci..

[32] Koebnick C., Mohan Y.D., Li X., Young D.R. (2015). Secular Trends of Overweight and Obesity in Young Southern Californians 2008-2013. J. Pediatr..

[33] Hamilton B.E., Martin J.A., Ventura S.J. (2011). Births: Preliminary data for 2010. Natl. Vital Stat. Rep..

[34] Olson C.M., Demment M.M., Carling S.J., Strawderman M.S. (2010). Associations Between Mothers’ and Their Children’s Weights at 4 Years of Age. Child. Obes..

[35] Yu Z.B., Han S.P., Zhu G.Z., Zhu C., Wang X.J., Cao X.G., Guo X.R. (2011). Birth weight and subsequent risk of obesity: A systematic review and meta-analysis. Obes. Rev..

[36] van Rossem L., Wijga A.H., Gehring U., Koppelman G.H., Smit H.A. (2015). Maternal Gestational and Postdelivery Weight Gain and Child Weight. Pediatrics.

[37] Kurspahić-Mujčić A., Mujčić A. (2020). Factors associated with overweight and obesity in preschool children. Med. Glas..

[38] Savva S.C., Tornaritis M., Chadjigeorgiou C., A Kourides Y., E Savva M., Panagi A., Chrictodoulou E., Kafatos A. (2005). Prevalence and socio-demographic associations of undernutrition and obesity among preschool children in Cyprus. Eur. J. Clin. Nutr..

[39] Janjua N.Z., Mahmood B., Islam M.A., Goldenberg R.L. (2012). Maternal and Early Childhood Risk Factors for Overweight and Obesity among Low-Income Predominantly Black Children at Age Five Years: A Prospective Cohort Study. J. Obes..

[40] Lanigan J., Barber S., Singhal A. (2010). Prevention of obesity in preschool children. Proc. Nutr. Soc..

[41] Baird J., Fisher D., Lucas P., Kleijnen J., Roberts H., Law C. (2005). Being big or growing fast: Systematic review of size and growth in infancy and later obesity. BMJ.

[42] Owen C.G., Martin R.M., Whincup P.H., Smith G.D., Cook D.G. (2005). Effect of Infant Feeding on the Risk of Obesity Across the Life Course: A Quantitative Review of Published Evidence. Pediatrics.

[43] Stunkard A.J., I Berkowitz R., Schoeller D., Maislin G., A Stallings V. (2004). Predictors of body size in the first 2 y of life: A high-risk study of human obesity. Int. J. Obes. Relat. Metab. Disord..

[44] Niinikoski H., Viikari J., Rönnemaa T., Helenius H., Jokinen E., Lapinleimu H., Routi T., Lagström H., Seppänen R., Välimäki I. (1997). Regulation of Growth of 7- to 36-Month-Old Children by Energy and Fat Intake in the Prospective, Randomized STRIP Baby Trial. Pediatrics.

[45] Hakanen M., Lagström H., Kaitosaari T., Niinikoski H., Näntö-Salonen K., Jokinen E., Sillanmäki L., Viikari J., Rönnemaa T., Simell O. (2006). Development of overweight in an atherosclerosis prevention trial starting in early childhood. The STRIP study. Int. J. Obes..

[46] Mo-Suwan L., Pongprapai S., Junjana C., A Puetpaiboon A. (1998). Effects of a controlled trial of a school-based exercise program on the obesity indexes of preschool children. Am. J. Clin. Nutr..

